# Dataset of Rat and Human Serum Proteomes Derived from Differential Depletion Strategies prior to Mass Spectrometry

**DOI:** 10.1016/j.dib.2020.105657

**Published:** 2020-05-06

**Authors:** Bharani Thangavelu, Gary H. Kamimori, Janice S. Gilsdorf, Deborah A. Shear, Angela M. Boutté

**Affiliations:** 1 Brain Trauma Neuroprotection Branch; 2 Blast Induced Neurotrauma Branch, Center for Military Psychiatry and Neuroscience, Walter Reed Army Institute of Research, 503 Robert Grant Avenue, Silver Spring, MD, USA-20910

**Keywords:** Serum, Depletion, Liquid Chromatography, Mass Spectrometry, Proteomics, Biomarker discovery

## Abstract

This article provides information regarding the effect of four common high abundant protein (albumin and immunoglobulins (Ig)) depletion strategies upon serum proteomics datasets derived from normal, non-diseased rat or human serum. After tryptic digest, peptides were separated using C_18_ reverse phase liquid chromatography-tandem mass spectrometry (rpLC-MS/MS). Peptide spectral matching (PSM) and database searching was conducted using MS Amanda 2.0 and Sequest HT. Peptide and protein false discovery rates (FDR) were set at 0.01%, with at least two peptides assigned per protein. Protein quantitation and the extent of albumin and Ig removal was defined by PSM counts. Venn diagram analysis of the core proteomes, derived from proteins identified by both search engines, was performed using Venny. Ontological characterization and gene set enrichment were performed using WebGestalt. The dataset resulting from each depletion column is provided.

Specifications table

**Table:** 

Subject	Biochemistry
Specific subject area	Proteomics, Biomarker Discovery, Mass Spectrometry, Serum Sample Processing
Type of data	Tables, Figures, excel files, graphs, mass spectrometry (.raw) files
How data were acquired	UltiMate 3000 RSLCnano, Orbitrap Fusion Lumos Tribrid mass spectrometer, Proteome Discoverer 2.2
Data format	Raw
	Analysed
Parameters for data collection	Depleted serum from human and rat collected and analyzed, in order to characterise the differential abundance of proteins.
Description of data collection	Comparative mass spectrometry-based proteomic profiling of serum proteome
Data source location	Data is collected and analysed at the
	Center for Military Psychiatry and Neuroscience
	Walter Reed Army Institute of Research
	503 Robert Grant Avenue
	Silver Spring, Maryland, USA
Data accessibility	Data are with this article and the MS/MS raw files have been deposited to the Mass Spectrometry Interactive Virtual Environment (MassIVE), a member of the Proteome Xchange consortium.
	Direct URL to data: ftp://massive.ucsd.edu/MSV000085008/
	doi:10.25345/C5GD7N

## Value of the data

• The data comprises workflow for proteomic analysis of depleted human and rat serum samples generated by a wide selection of commercially available kits that might be a useful for other researchers to select the method of choice according to the target of interest.
• The dataset includes comparison of protein content derived from spectral count data as defined by MS Amanda and Sequest HT search engines and peptide-spectrum match (PSM) output that might be a useful for other researchers for optimization of search engines and post processing approaches to maximize peptide and protein identification for high-resolution mass data.
• The dataset includes serum proteomes for non-injured rats and healthy human subjects that might be a useful for other researchers for baseline or control dataset, reflective of a normal or healthy conditions, for which discovery of putative biomarkers may be compared.

## 1 Data

**Fig. 1:**
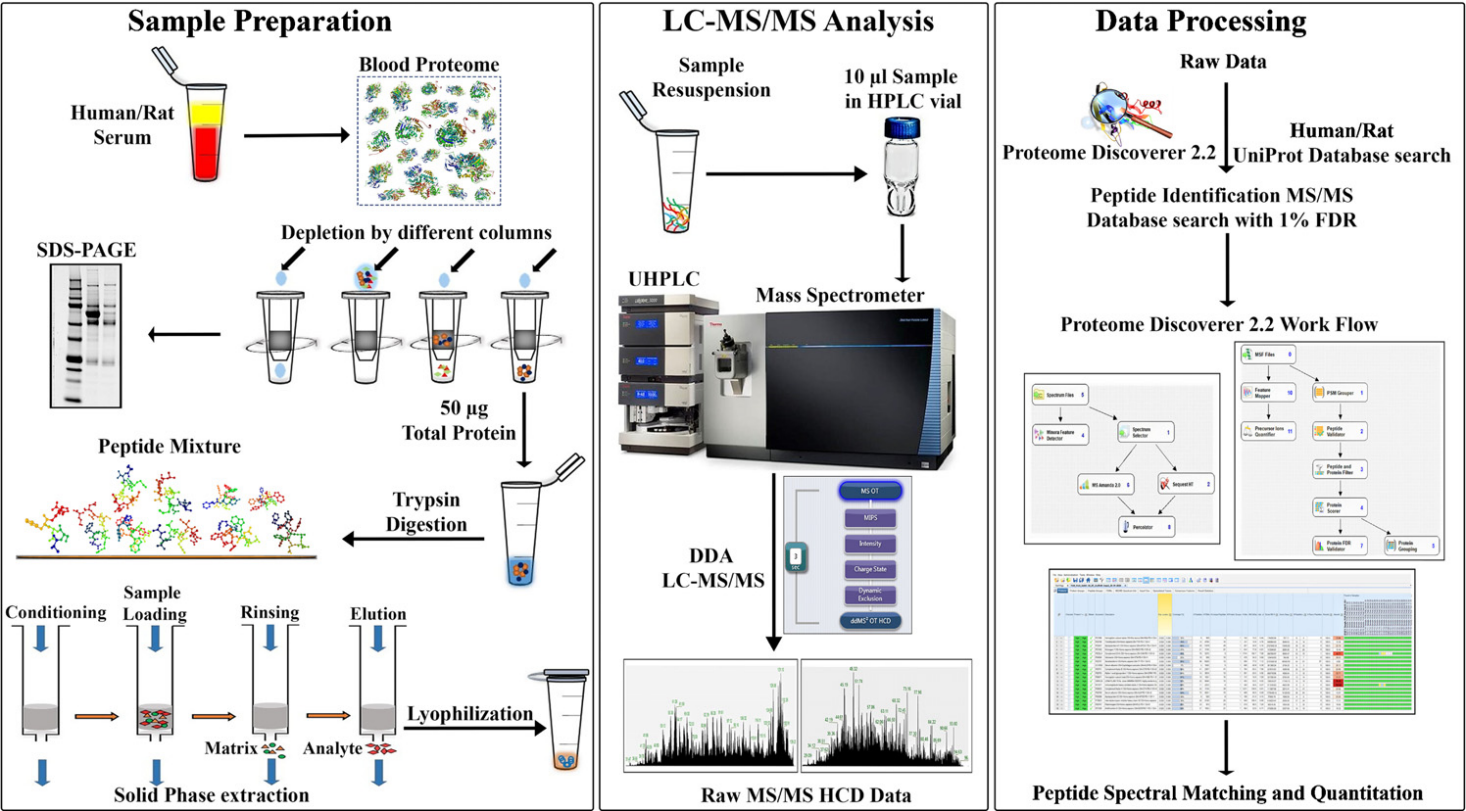
Schematic Representation of Serum Preparation, rpLC-MS/MS analysis, and data processing.

The work flow for sample preparation, data collection, processing and analysis are indicated (Fig. 1) for four commercially available depletion columns (Supplementary Table 1).

The total number of PSMs and extent of albumin (Table 1) or Ig (Table 2) removal was determined in depleted human or rat serum samples. Serum proteins that were detected for each condition are displayed with for human (Supplementary Tables 2 A-E) and rat serum (Supplementary Tables 3 A-E). The accession number, protein name, description, PSMs, and q-values, as well as information regarding the number of total or unique peptides are indicated for each proteome as derived from each search engine, Sequest HT or MS Amanda.

**Table 1:** Albumin Content in Serum before and after Depletion. The table shows the total number of PSMs from MS Amanda 2.0 and Sequest HT detected in crude or depleted serum using four different columns for both human (left) and rat (right) serum.

Column name	Database Search Engine	Human					Rat				
		Total PSMs	Albumin Specific PSMs	Replicate CV (%)	Albumin PSMs (% of Total PSMs)	Depletion Efficiency (% of Albumin PSMs in Crude Serum)	Total PSMs	Albumin Specific PSMs	Replicate CV (%)	Albumin PSMs (% of Total PSMs)	Depletion Efficiency (% of Albumin PSMs in Crude Serum)
Undepleted/Crude Serum	MS Amanda 2.0	13675	4787	1.8	35.0	N/A	9309	1733	1.6	18.6	N/A
	Sequest HT	19948	7470	2.0	37.4	N/A	13812	2676	1.8	19.4	N/A
Top 12™ Abundant Protein Depletion	MS Amanda 2.0	7256	232	8.5	3.20	95.2	6316	551	3.2	8.72	68.2
	Sequest HT	9880	391	8.8	3.96	94.8	9491	1089	1.9	11.5	59.3
PureProteome™ Albumin/IgG Magnetic Beads	MS Amanda 2.0	11732	270	5.0	2.30	94.4	6708	254	5.0	3.79	85.3
	Sequest HT	18500	503	3.3	2.72	93.3	9883	517	3.3	5.23	80.7
AlbuSorb™ PLUS	MS Amanda 2.0	10211	1776	2.1	17.4	62.9	6544	291	6.0	4.45	83.2
	Sequest HT	15621	3192	2.0	20.4	57.3	9960	686	2.5	6.89	74.4
Seppro® Rat Spin	MS Amanda 2.0	9147	2170	1.0	23.7	54.7	7907	96	5.6	1.21	94.5
	Sequest HT	13355	3866	0.5	28.9	48.2	11017	220	6.8	2.00	91.8

N/A= Not applicable.

**Table 2:** Ig Content in Serum before and after Depletion. The table shows the total number of PSMs from MS Amanda 2.0 and Sequest HT detected in crude or depleted serum using four different columns for both human (left) and rat (right) serum.

Column name	Database Search Engine	Human Serum				Rat Serum			
		Total PSMs	Ig Specific PSMs	Ig PSMs (% of Total PSMs)	Depletion Efficiency (% of Ig PSMs in Crude Serum)	Total PSMs	Ig Specific PSMs	Ig PSMs (% of Total PSMs)	Depletion Efficiency (% of Ig PSMs in Crude Serum)
Undepleted/Crude Serum	MS Amanda 2.0	13675	2786	20.4	N/A	9309	1025	11.0	N/A
	Sequest HT	19948	3897	19.5	N/A	13812	1162	8.41	N/A
Top 12™ Abundant Protein Depletion	MS Amanda 2.0	7256	42	0.58	98.5	6316	400	6.33	61.0
	Sequest HT	9880	102	1.03	97.4	9491	649	6.84	44.1
PureProteome™ Albumin/IgG Magnetic Beads	MS Amanda 2.0	11732	367	3.13	86.8	6708	592	8.83	42.2
	Sequest HT	18500	598	3.23	84.7	9883	899	9.10	22.6
AlbuSorb™ PLUS	MS Amanda 2.0	10211	1508	14.8	45.9	6544	478	7.30	53.4
	Sequest HT	15621	3045	19.5	21.9	9960	844	8.47	27.4
Seppro® Rat Spin	MS Amanda 2.0	9147	908	9.93	67.4	7907	251	3.17	75.5
	Sequest HT	13355	1376	10.3	64.7	11017	414	3.76	64.4

N/A= Not applicable.

**Fig. 2:**
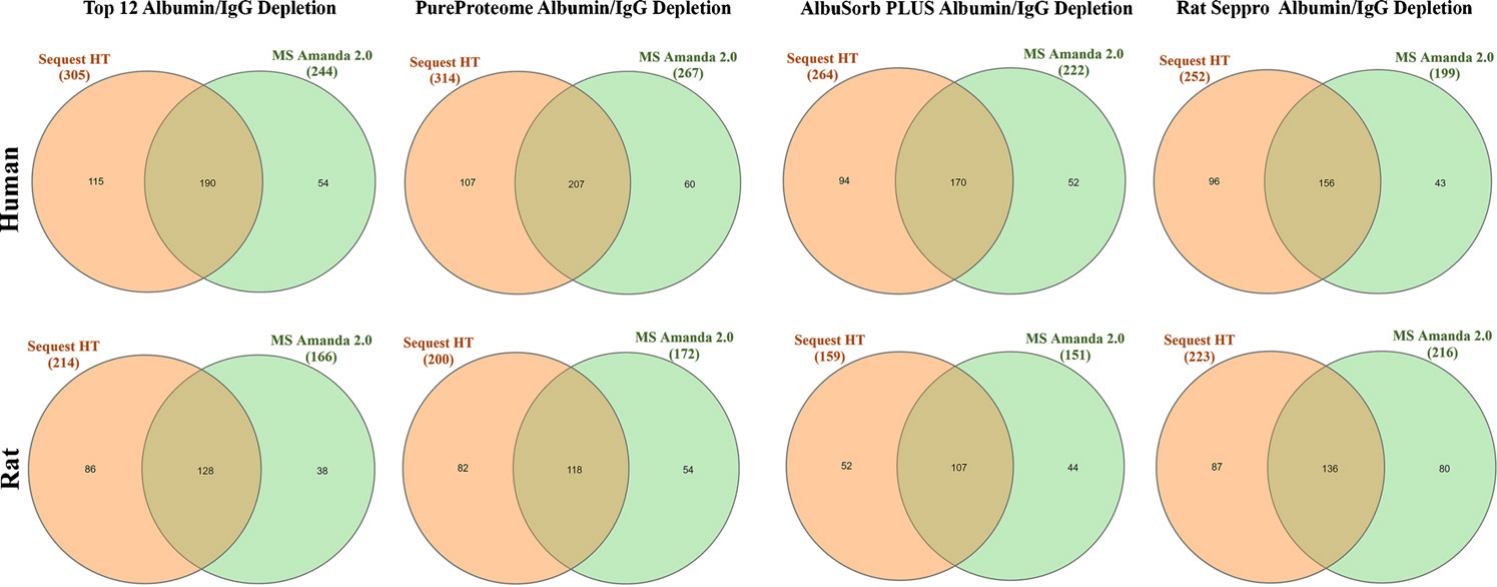
Venn Diagrams of Serum Proteins Identified by Sequest and MS Amanda after Depletion. Venn diagrams are shown for proteins with the same Uniprot ID detected in either human (top) or rat (bottom) serum samples by Sequest HT (left, orange) and MS Amanda (right, green) after depletion with Top12, PureProteome, Albusorb, and Seppro Columns.

**Fig. 3:**
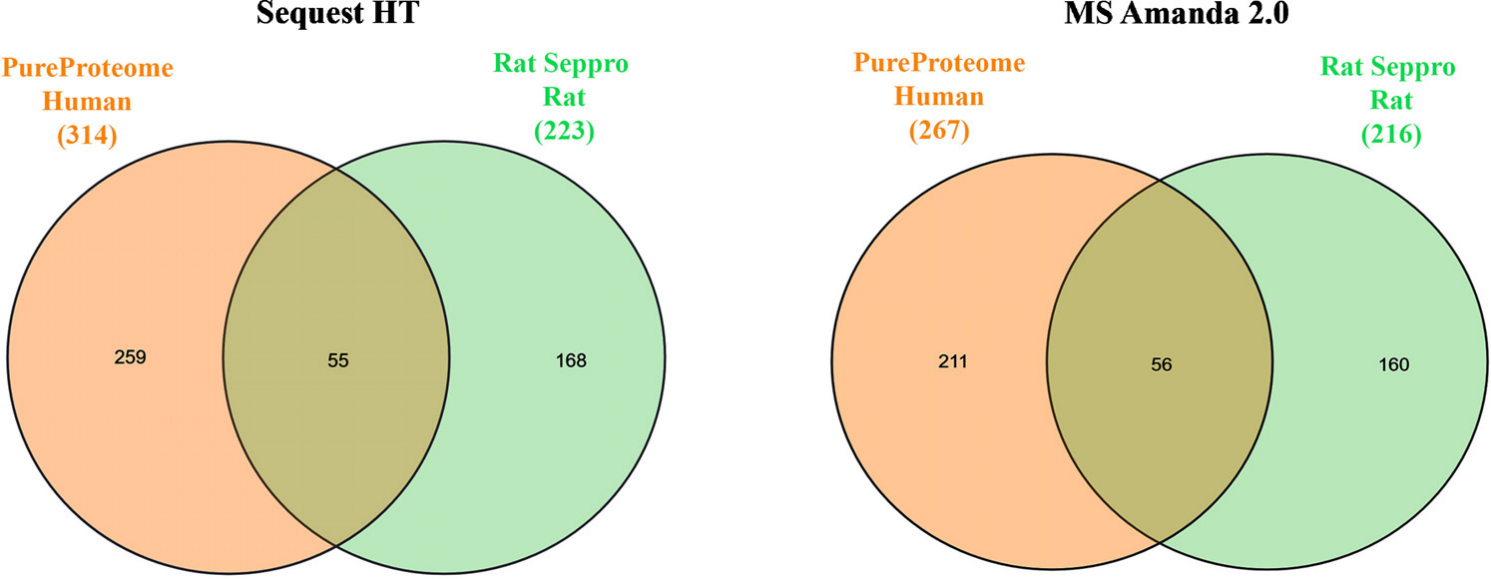
Venn Diagrams of Serum Proteins Derived from Columns with High Recovery and Low Albumin and Ig Content. Venn diagrams are shown for proteins with the same protein name detected in either human (left, orange) or rat (right, green) serum samples by Sequest HT (left) and MS Amanda (right) after depletion with PureProteome, and Seppro Columns, respectively.

**Fig. 4:**
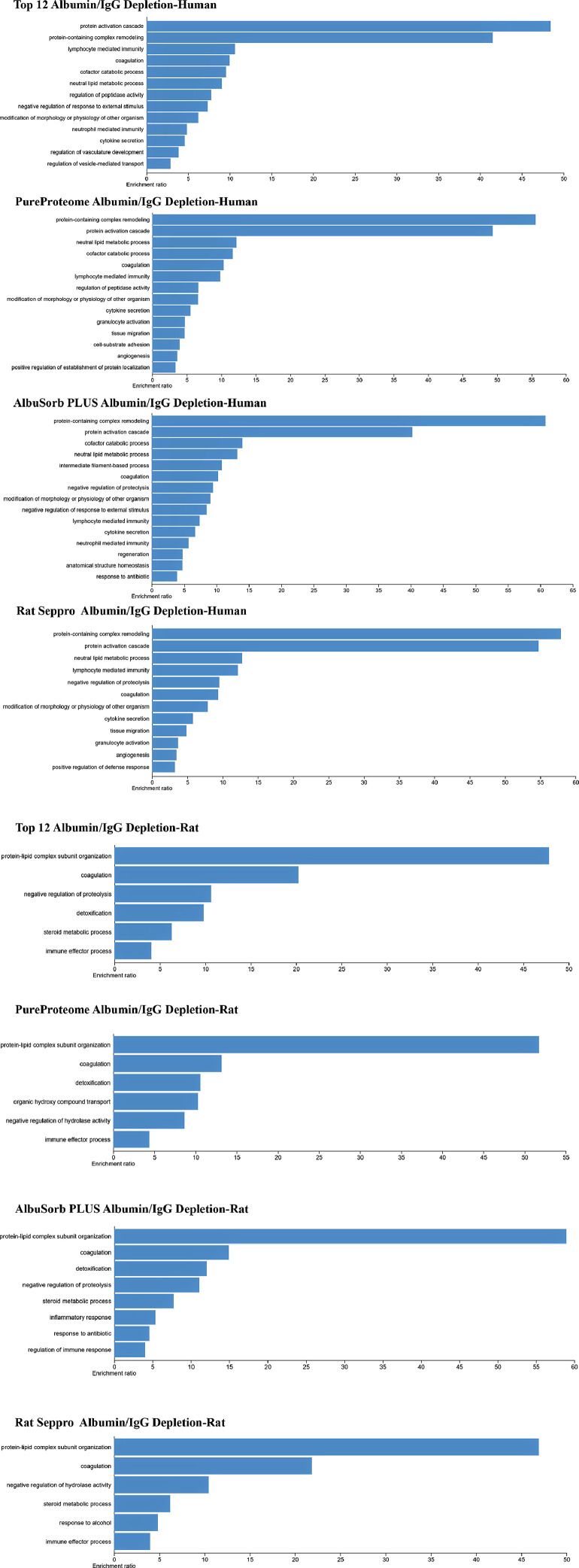
Impact of Depletion Strategies upon Gene Set Enrichment. Over-represented gene set categories derived from depleted proteomes of (A) human, or (B) rat serum. The ratio of enrichment is shown (Benjamini-Hochberg, FDR ≤ 0.05).

The number of proteins from each depletion column and search engine were compared using Venn diagram analysis (Fig. 2). Next, the concordance among the human and rat serum proteomes was compared. The serum proteome from each species was derived from column that led to greatest number of unique, non-redundant, protein identifications (Fig. 3). Lastly, the core proteome was defined by the list of proteins identified in both Sequest and MS Amanda for each species and column used. This protein list was then used to define the extent to which depletion strategies affected overall characterization of the serum proteome defined by WEB-based Gene SeT AnaLysis Toolkit (WebGestalt) based on biological function (Fig. 4 A-B).

## 2 Experimental Design, Materials, and Methods

### 2.1 Serum Preparation and Depletion

Pooled serum from either healthy males, aged 20-30 (n = 10) or male Sprague Dawley rats (n=10) (BioIVT, Baltimore, MD) were thawed on ice, centrifuged at 1,500 x g for 10 min at 4° C, then split into aliquots and stored at -80° C until biochemical analysis. For column-based depletion, serum samples were thawed on ice then filtered using 0.45 µm cellulose acetate microspin columns (Sigma-Aldrich Inc., St. Louis, MO, USA). Twelve microliters of sera was used for Top 12™ Abundant Protein Depletion Spin Columns (Pierce Biotechnology, Rockford, lL, USA), and 25uL each was used for depletion of sera with either PureProteome™ Albumin/IgG Magnetic Beads (Millipore Sigma, Burlington, MA, USA), AlbuSorb™ PLUS (Biotech Support Group, Monmouth Junction, NJ, USA), or Seppro® Rat Spin Columns (Sigma-Aldrich Inc., Saint. Louis, MO, USA). All columns are reported to remove albumin and IgGs, but the Top 12™ Abundant Protein Depletion Column also removes ten additional proteins, including Alpha-1-Acid glycoprotein, Alpha-1-Antitrypsin, Alpha-2-Macroglobulin, Apolipoproteins A-I and A-II, Fibrinogen, Haptoglobin, IgA, IgM, and Transferrin. The Seppro® Rat Spin Column removes five additional proteins, namely Alpha-1-Antitrypsin, Fibrinogen, Haptoglobin, IgM as well as Transferrin. Although this column is intended for the rat based on the manufacturer's instructions, it was is reported to have cross reactivity with high abundant proteins in human sera [1]. Depletion procedures were performed at room temperature (RT, 20-25° C) according to manufacturer's instructions. Eluted, depleted serum was immediately stored at 4^o^ C until further analysis.

### 2.2 Protein Assays

Total protein content of depleted sera was determined using the microBCA protein assay according to the manufacturer's instructions (Pierce Biotechnology, Rockford, lL, USA).

### 2.3 Tryptic Digest

Depleted serum containing 50 µg of protein denatured and reduced with 8M urea supplemented with 1M DTT, shaking the sample at 500 RPM using a 37^o^ C heated shaker for 45 minutes. Samples were alkylated with iodoacetamide (IAA) (50mM final concentration, Sigma-Aldrich Inc., St. Louis, MO, USA) in the dark for 45 minutes. Samples were diluted to ≤ 1M urea then supplemented with 1 mL of 50 mM NH_4_HCO_3_, pH 8.0), and with 2-2.5 µL of 6N NaOH to adjust the pH to 8.5 – 9.0. Samples were then digested by adding 2 µg of Trypsin Gold (Promega, Madison, WI, USA) for 16-18 hours, shaking at 500 RPM, at 37^o^ C. Digestion was terminated with 20 µL formic acid (Sigma-Aldrich Inc., St. Louis, MO, USA). The final pH was adjusted to 2.5 – 3.5 using 6N HCl.

### 2.4 Solid Phase Extraction

Empore™ Solid Phase Extraction Cartridges (3M, St. Paul, MN, USA) were used to remove debris from the sample prior to analysis. Briefly, cartridges were washed twice with 1 mL Activation Buffer (80% acetonitrile, 20% water and 0.1 % trifluoroacetic acid (TFA)) then twice with 1 mL Wash Buffer (95% water, 5% acetonitrile, and 0.1 % TFA). Digested serum-derived protein samples were added to the column prior to centrifugation at 1,500 g, at RT. Bound peptides were washed twice with 1 mL Wash Buffer. Positive pressure centrifugation was applied at RT for processing liquids through disk cartridges (EBA 20, Hettich Zentrifugen, Tuttlingen, Germany) at 1500 x g. Eluted peptides were collected with 1 mL Activation Buffer into LoBind microcentrifuge tubes (Eppendorf, Hamburg, Germany), then dried (Savant TM, SPD131DDA SpeedVac, Thermo Fisher Scientific, Waltham, MA, USA) for 3-4 hours at RT. Lyophilized samples were stored at -80° C until rpLC-MS/MS analysis.

### 2.5 Mass Spectrometry

Lyophilized sera-derived peptides were thawed on ice for 30 minutes and reconstituted in 100 µL of sterile, proteomics grade peptide sample buffer (95% water, 5% acetonitrile (ACN), and 0.1 % formic acid (FA)). Samples were filtered using 0.45 µm cellulose acetate microspin filters which was pre-washed with sample buffer. Thereafter, ten µL was transferred to glass HPLC vials (Waters, Milford, MA, USA). Rp-LC was performed using a binary high-pressure gradient pump UltiMate 3000 RSLCnano system with a Dionex WPS-3000 autosampler (Thermo Fisher Scientific, Germering, Germany) coupled to an EASY-Spray column. Data acquisition and gradient control was performed with Chromeleon, Version 7.0 (Dionex, Sunnyvale, CA, USA). Human sera peptides were concentrated and washed on a trapping pre-column (Acclaim PepMap C_18_, 75 µm × 2 cm nanoViper, 3 µm, 100 Å, Thermo Fisher Scientific), then separated using a C_18_ reversed phase column (Acclaim PepMap RSLC C_18_, 50 µm × 15 cm nanoViper, 2 µm, 100 Å, Thermo Fisher Scientific) with linear gradient of 150 min from 2-95% of Eluent B (0.1% formic acid in 100% acetonitrile) in Eluent A (0.1% formic acid in 100% Water) at a flow rate of 300 nL/min. Rat sera peptide mixtures were fractionated on a RSLC C_18_ column, 25 cm × 75 µm nanoViper, 2 µm, 100Å, using a linear gradient of 150 min from 2-95% of Eluent B. MS/MS analysis was performed using an Orbitrap Fusion Lumos Tribrid mass spectrometer (Thermo Fisher Scientific, Waltham, MA, USA) in data-dependent positive ion mode with Xcalibur, v.1.0.2.65 SP2 (Thermo Fisher Scientific, Bremen, Germany). The scan range was 350−1600 m/z for precursors (MS1), followed by charge-state determination and higher-energy collisional dissociation (HCD) scan carried out on the ten most intense ions, and data collection in profile mode. Monoisotopic peak determination, charge-state screening, and data dependent dynamic exclusion were enabled, with exclusion of non-assigned peptides, an intensity threshold of 3 × 10^4^, a repeat count of two, and an exclusion duration of 60s for ions ±10 ppm of the parent ion mass. The automatic gain control (AGC) settings were 4 × 10^5^ and 2 × 10^5^ ions for survey, and HCD modes, respectively. Scan times were set at 50 for survey mode and at 100 ms for HCD mode. For HCD, collision energy were set at 35%. Quadrupole isolation mode and the Orbitrap detector was used for both for survey mode (resolution = 120, 000) and HCD mode (resolution = 15, 000). All data was acquired is centroid mode and all runs were carried out in triplicate.

### 2.6 Database Search and Label Free Quantitation

RpLC-MS/MS data was analyzed using a pipeline implemented in Proteome Discoverer, version 2.2 (ThermoFisher Scientific, Bremen, Germany). Mass spectrometry .raw files were searched with MS Amanda (version 2.0) and Sequest HT against human or rat databases from UniProtKB/SwissProt (release 2018-06) with the following parameters: two tryptic missed cleavages; precursor mass tolerance ≤10 ppm; MS/MS mass tolerance ≤ 0.02 Da; charge states of + 2, + 3, and + 4; cysteine carbamidomethylation (+ 57.021 Da) as static modification, and methionine oxidation (+15.995 Da) as dynamic modification. Protein and peptide validation (FDR < 0.01%) was determined using Percolator. Label-free quantification was conducted using all peptides with a q-value of ≤ 0.01 and a peptide rank ≥ 1.

### 2.8 Data Analysis

Protein abundance was determined by count the number of peptide-spectrum matches (PSM). Efficiency of albumin and Ig depletion was calculated as the number of peptide-spectrum matches detected after before and depletion. Due to the diversity in Ig proteins, spectral counting (e.g. PSMs) of Ig proteins and their subclasses that were expected to be depleted across all columns per the manufacturers’ specifications were included in comparative analysis per species. Uniprot/SwissProt accession IDs for human Ig proteins gamma 1-4 (P0DOX5, 49.3 kDa; P01859 35.9, kDa; P01860 41.3 kDa; and P01861, 35.9kDa), as well as Ig light chains from Ig kappa (P0DOX7, 23.4 kDa) and lambda chains (P0DOX8, 22.8 kDa) were analyzed. Similarly, rat IgG heavy chain was identified based on detection of subclasses Gamma 1 and 2 A-C (P20759, P20760, P20761 and P20762, =36 kDa). Rat light chains were identified as Ig kappa (P01835, 11.6 kDa and P01836, 11.7 kDa) and lambda chains (P20767, 11.3 kDa). Comparisons between proteomes per search engine were defined using Venn diagrams generated with Venny 2.0 (http://bioinfogp.cnb.csic.es/tools/venny/index.html). Proteins identified in both search engines was defined as the “core proteome” and used for ontological characterization based on biological function using WebGestalt [2]. Hypergeometric test for enrichment analysis (Benjamini-Hochberg, FDR ≤0.05) with a minimum of five proteins was applied. The ratio of enrichment is indicated for each category.

## Disclaimer

Material has been reviewed by the Walter Reed Army Institute of Research. There is no objection to its presentation and/or publication. The opinions or assertions contained herein are the private views of the author, and are not to be construed as official, or as reflecting true views of the Department of the Army or the Department of Defense. No human subjects or animals were used in the research. Human and rat sera were purchased from commercially available repository (BioIVT, Baltimore, MD). Images of the Lumos Orbitrap mass spectrometer were used with permission (Thermo Fisher Scientific, Waltham, MA).

## Declaration of Competing Interest

The authors declare that they have no known competing financial interests or personal relationships that could have appeared to influence the work reported in this paper.

## Appendix Supplementary materials

Image, application 1

Image, application 2
